# Small molecules that inhibit the late stage of Munc13-4–dependent secretory granule exocytosis in mast cells

**DOI:** 10.1074/jbc.RA117.001547

**Published:** 2018-04-03

**Authors:** Stephen Bruinsma, Declan J. James, Melanie Quintana Serrano, Joseph Esquibel, Sang Su Woo, Elle Kielar-Grevstad, Ellen Crummy, Rehan Qurashi, Judy A. Kowalchyk, Thomas F. J. Martin

**Affiliations:** From the Department of Biochemistry, University of Wisconsin, Madison Wisconsin 53706

**Keywords:** membrane fusion, mast cell, exocytosis, small molecule, secretion, inhibitor, intracellular trafficking, Munc13-4

## Abstract

Ca^2+^-dependent secretory granule fusion with the plasma membrane is the final step for the exocytic release of inflammatory mediators, neuropeptides, and peptide hormones. Secretory cells use a similar protein machinery at late steps in the regulated secretory pathway, employing protein isoforms from the Rab, Sec1/Munc18, Munc13/CAPS, SNARE, and synaptotagmin protein families. However, no small-molecule inhibitors of secretory granule exocytosis that target these proteins are currently available but could have clinical utility. Here we utilized a high-throughput screen of a 25,000-compound library that identified 129 small-molecule inhibitors of Ca^2+^-triggered secretory granule exocytosis in RBL-2H3 mast cells. These inhibitors broadly fell into six different chemical classes, and follow-up permeable cell and liposome fusion assays identified the target for one class of these inhibitors. A family of 2-aminobenzothiazoles (termed benzothiazole exocytosis inhibitors or bexins) was found to inhibit mast cell secretory granule fusion by acting on a Ca^2+^-dependent, C2 domain–containing priming factor, Munc13-4. Our findings further indicated that bexins interfere with Munc13-4–membrane interactions and thereby inhibit Munc13-4–dependent membrane fusion. We conclude that bexins represent a class of specific secretory pathway inhibitors with potential as therapeutic agents.

## Introduction

Ca^2+^-triggered secretory granule (SG)^7^ fusion with the plasma membrane is the final step in the regulated secretory pathway for protein and peptide secretion. Diverse secretory cell types employ the regulated pathway for the secretion of mucins, proteases, inflammatory mediators, biogenic amines, neuropeptides, and peptide hormones. The core machinery for Ca^2+^-triggered SG–plasma membrane fusion characterized in neural, neuroendocrine, and myeloid secretory cells consists of SG arginine (R)-soluble *N*-ethylmaleimide-sensitive factor attachment protein receptor (SNARE) proteins that form fusion-competent complexes with plasma membrane glutamine(Q)-SNARE proteins (1). SNARE protein complex assembly involves the concerted actions of Rab, Sec1/Munc18, and Munc13/CAPS protein family members to enable SG docking and priming (2, 3). Final Ca^2+^-triggered fusion steps utilize SG-localized synaptotagmins or other Ca^2+^ sensors (4). Constitutive secretory pathways operating in parallel utilize distinct Rab, SNARE, Sec1/Munc18, and tethering/priming factors such as the exocyst (5). Although there are many protein targets in the final steps of regulated or constitutive vesicle exocytosis, few small molecule inhibitors have been identified (see “Discussion”) (6, 7). Mechanism-selective small-molecule inhibitors would be useful for cell biological and physiological studies.

There are potential therapeutic applications for inhibitors of SG exocytosis for a number of secretory cell types, including mast cells. Mast cells store pre-formed vasoactive and pro-inflammatory mediators such as histamine, serotonin, tumor necrosis factor α (TNFα), and proteases in SGs (8, 9). Mediator release occurs by Ca^2+^-dependent SG exocytosis (termed degranulation) in response to allergens, pathogens, and cytokines acting at the cell surface. Mast cell mediators play roles in allergic responses, asthma, autoimmunity, and inflammation (9). Therapies for mast cell hypersecretion have targeted upstream components in signaling pathways, including FcϵRI IgE receptors, Syk, phosphatidylinositol 3-kinase, or Ca^2+^ release–activated channel (CRAC) channels (10). Alternatively, released mediators have been targeted by specific antagonists such as protease inhibitors, antihistamines, or TNFα antibodies (10). However, mechanism-specific inhibitors that directly target Ca^2+^-triggered degranulation have yet to be characterized (11). Such small-molecule inhibitors could provide new therapies for mast cell–linked pathologies.

Phenotypic assays for mast cell degranulation have been reported for high-throughput screening of chemical compound diversity libraries, and several small-molecule inhibitors have been detected (12, 13). However, it has been difficult to characterize the specific protein targets of those small molecules (14). This work utilized a series of assays to identify protein targets of inhibitors that act at final steps in Ca^2+^-dependent SG exocytosis in mast cell-like RBL-2H3 cells. A high-throughput fluorescence-based assay was used to screen an ∼25,000-compound diversity library for inhibitors of ionomycin-induced degranulation. Follow-up assays with a permeable cell assay and a SNARE-dependent liposome fusion assay discovered inhibitors that target a protein required for mast cell SG exocytosis. A family of 2-aminobenzothiazole compounds (termed bexins) was characterized as inhibitors of Munc13-4, an essential Ca^2+^-dependent priming factor for Ca^2+^-triggered degranulation in mast cells. Additional studies suggest that bexins interfere with membrane interactions required for Munc13-4 function in Ca^2+^-dependent membrane fusion.

## Results

### High-throughput screen for small-molecule inhibitors of RBL-2H3 cell degranulation

**Figure 1.**
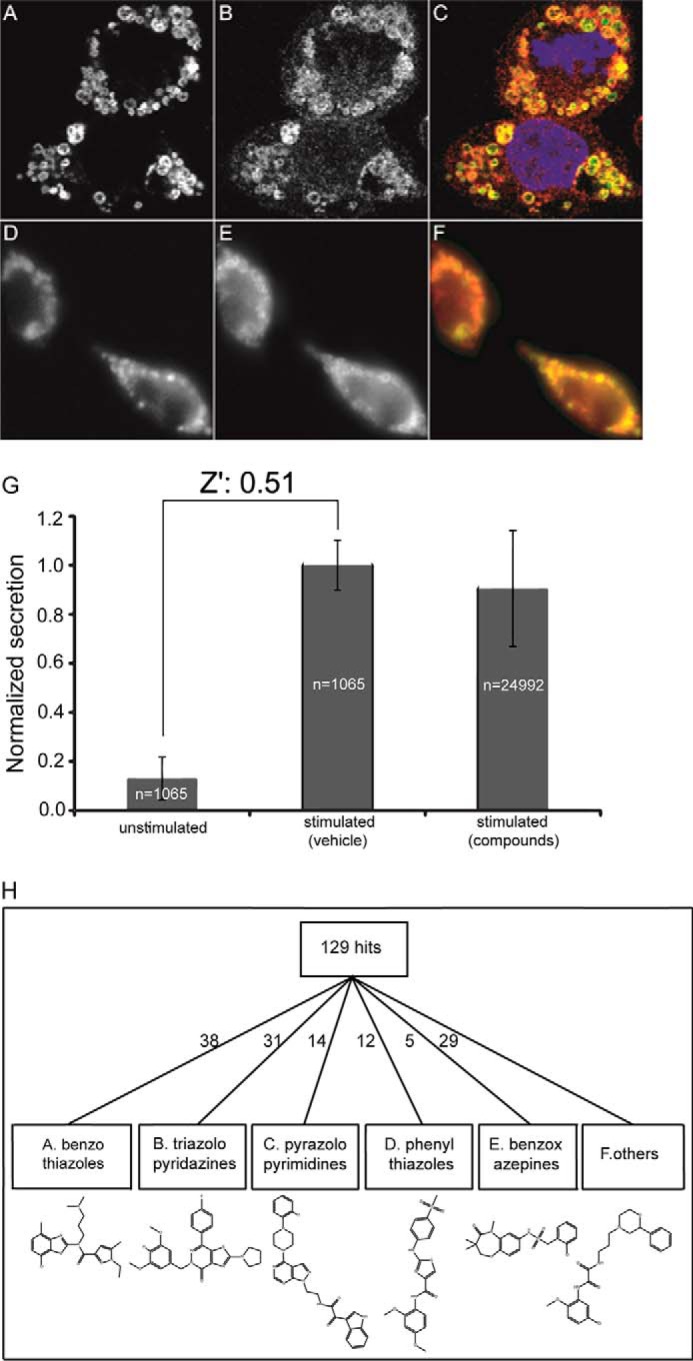
**Screening strategy and summary of inhibitors of secretion.** *A–F*, ANF-EGFP (*A* and *D*) localized to secretory granules detected by VMAT2 (*B*) and RMCP2 (*E*) immunofluorescence. Merge panels (*C* and *F*) indicate co-localization. *G*, ANF-EGFP secretion was stimulated by ionomycin treatment. EFGP fluorescence was measured in overlying medium and detergent-solubilized cells after 15 min of incubation without or with ionomycin. Maximal percent secretion was normalized to 1.0. Values shown (mean ± S.D.) are from a screen for controls without ionomycin (1065 wells), ionomycin-treated without compounds (1065 wells), or from ionomycin-treated (24,992 wells) with 4 μm compounds. Hits in the screen reduced secretion by ≥50% at 4 μm. *H*, DataWarrior was used to sort 129 confirmed hits into five major classes (A–E) based on ring structure plus a diverse sixth class (F). The representative class structures shown correspond to F5128-0085 (A), F2927-0504 (B), F2902-0553 (C), F5448-0530 (D), F2297-0090 (E), and F5024-0157 (F).

To identify small-molecule inhibitors of degranulation, a high-throughput assay for regulated SG exocytosis in RBL-2H3 mast cells was developed. A fluorescent fusion protein, atrial natriuretic factor–enhanced GFP (ANF-EGFP) was expressed as SG cargo so that a cytoplasmic Ca^2+^ rise elicited its release by Ca^2+^-triggered SG exocytosis. Stably expressed ANF-EGFP co-localized with the SG membrane amine transporter protein VMAT2 and the endogenous SG protease RMCP2 (Fig. 1, *A–F*). The Ca^2+^ ionophore ionomycin stimulated the secretion of ANF-EGFP from RBL-2H3 cells (Fig. 1*G*). Stimulation by ionomycin bypasses upstream signaling components and cellular Ca^2+^ entry mechanisms, so the assay focused on steps in Ca^2+^-dependent SG exocytosis downstream of Ca^2+^ entry.

The ANF-EGFP secretion assay was adapted to robotic liquid transfer in 384-well plates, and an ∼25,000-compound diversity library (Life Chemicals II) was screened. Cells were pretreated with each test compound (4 μm each) in DMSO (0.1% final) for 15 min before stimulation with 2 μm ionomycin for 15 min. Secreted ANF-EGFP in the medium was removed, and cells were solubilized in detergent to measure secreted and cell-retained fluorescence, respectively. Percent secretion was calculated as (medium fluorescence/(medium + cellular fluorescence) × 100%). 32 wells on each 384-well plate received 0.1% DMSO, and 16 of these were stimulated with ionomycin for calculating Z' values per plate.

The average percentages of ANF-EGFP secretion for unstimulated and stimulated controls were 5% and 41%, respectively. The average Z' value per plate was 0.51 (Fig. 1*G*), indicating a good signal for the identification of inhibitors (15). Inhibitors in the primary screen were identified as compounds that reduced secretion by more than 4 S.D. from the mean. These were retested at three concentrations (0.4, 1.3, and 4.0 μm), and 129 compounds were confirmed to inhibit stimulated degranulation by more than 50% at 4 μm. The inhibitory compounds were clustered into classes based on major ring systems as analyzed in DataWarrior using a combination of the FragFp library and International Union of Pure and Applied Chemistry nomenclature from the ChemAxon JChem Suite. Five major classes (A–E) and a diverse class (F) of inhibitors were identified (Fig. 1*H*).

**Figure 2.**
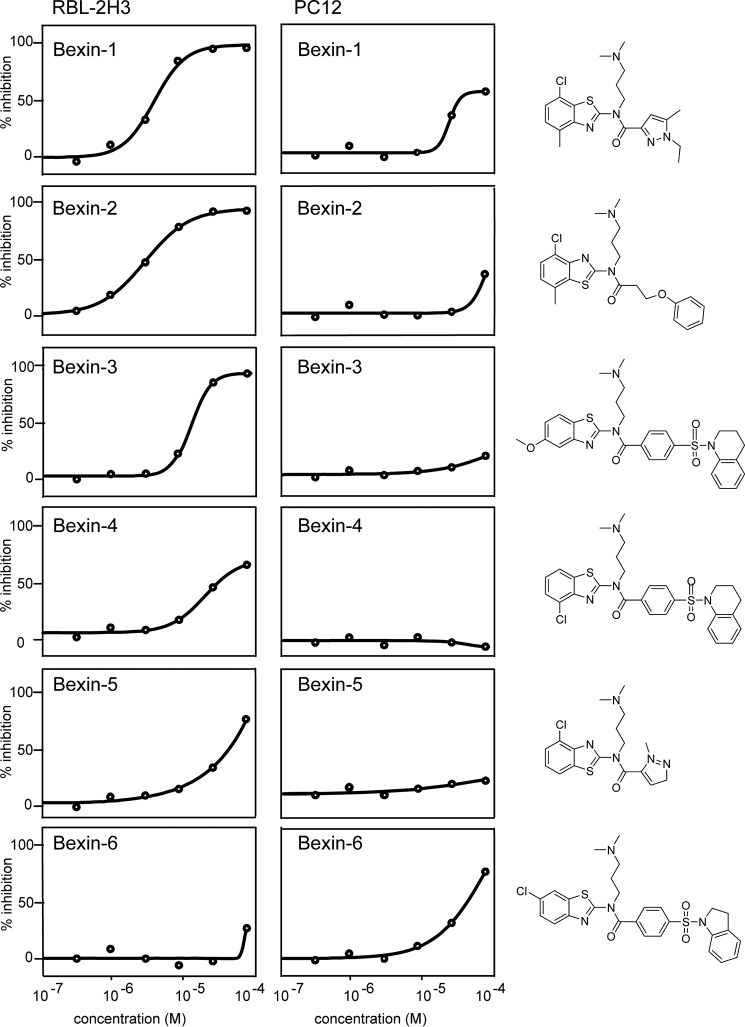
**Apparent cell specificity of 2-aminobenzothiazole inhibitors.** Six members of the 2-aminobenzothiazole class (class A) were tested for the inhibition of ionomycin-stimulated ANF-EGFP secretion in RBL-2H3 cells (*left column*) or PC12 cells (*right column*). Values are the mean with S.D. values that were within 20% of the mean (*n* = 3). Structures of the indicated compounds are shown in the *right panel*. For RBL-2H3 cells (*top* to *bottom*), compounds bexin-1 (F5128-0085), bexin-2 (F2019-1453), bexin-3 (F2019-1958), bexin-4 (F2019-1450), bexin-5 (F5085-0061), and bexin-6 (F2019-0631) exhibited IC_50_ values of 2.3 μm, 1.8 μm, 6.1 μm, 27.5 μm, 26.7 μm, and undetermined, respectively, as assessed by SigmaPlot.

38 of the 129 strong inhibitors were 2-aminobenzothiazoles (class A). Based on subsequent results, these were termed bexins (benzothiazole exocytosis inhibitors). Two of the strongest 2-aminobenzothiazoles, bexin-1 and -2, exhibited IC_50_ values of ∼3 μm (Fig. 2, *left column*). By examining the compound library, we also identified compounds with similar 2D structures that were not inhibitory in the primary screen, such as bexin-5 and -6; these were at least ∼20-fold less effective than bexin-1 (Fig. 2, *left column*). Despite very similar 2D structures for bexin-1 and -5, energy minimizations performed in SYBYL and the PRODRG server (16) revealed that these compounds exhibit different 3D structures. The benzothiazole ring is extended in the same plane as the pyrazole ring in bexin-1, whereas these ring systems adopt a more compact chair-like configuration in bexin-5. In subsequent studies, bexin-5 was used as an inactive control for inhibitory bexin-1. Inhibitors in other classes (B–F) were shown to be of less interest in subsequent studies (Fig. 4).

### Cell type specificity for inhibition by bexins

Bexin-1 inhibited secretion in RBL-2H3 cells but was much less potent in inhibiting secretion in a parallel assay employing PC12 neuroendocrine cells (Fig. 2, *right column*). Similarly, bexin-2, and -3 inhibited secretion in RBL-2H3 cells but exhibited reduced inhibition in PC12 cells, even at 80 μm (Fig. 2, *right column*). By contrast, bexin-6, which was inactive in RBL-2H3 cells, was weakly inhibitory in PC12 cells. One explanation for different results in two cell types might be differences in membrane permeability. However, this was rendered unlikely in studies with permeable cells where drug potencies were similar to those in intact cells. Alternatively, different secretory cells might utilize different protein isoforms in regulated SG exocytosis. In this case, RBL-2H3 cells, but not PC12 cells, might express a specific protein isoform that is a target for bexins (see “Discussion”). Overall, the results shown in Fig. 2 indicate that bexins are not general inhibitors of the regulated secretory pathway but may target specific protein isoforms that are essential for regulated SG exocytosis in RBL-2H3 mast cells.

### Specificity of inhibitors for mast cell exocytosis

**Figure 3.**
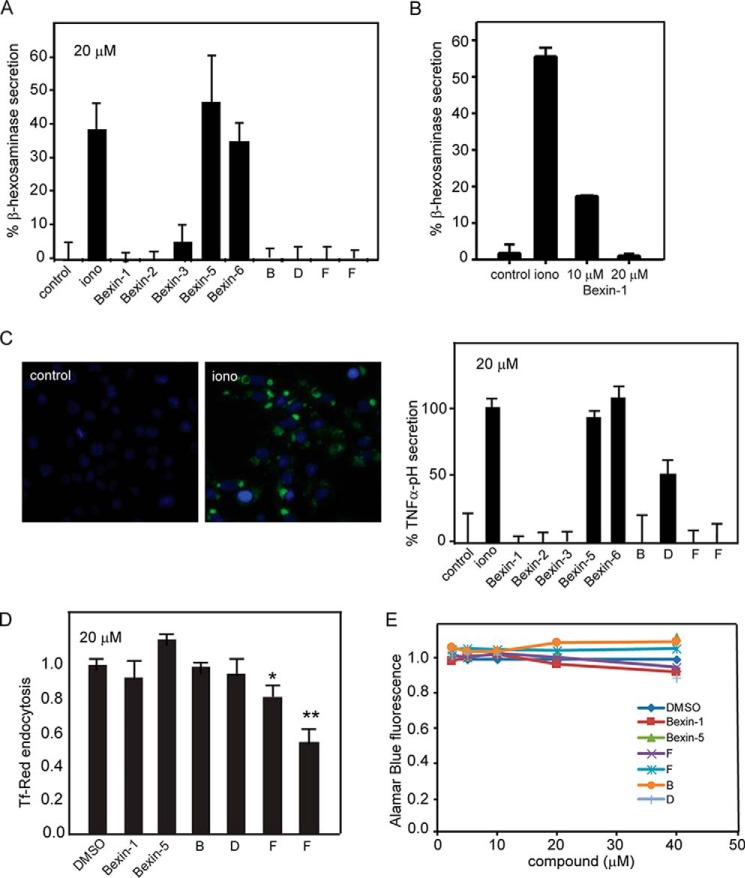
**Compound testing in orthogonal and membrane trafficking assays.** *A*, β-hexosaminidase secretion from RBL-2H3 cells in 15 min was determined for untreated (*control*) or ionomycin (*iono*)-treated cells without or with the indicated compound at 20 μm provided 15 min prior to ionomycin stimulation. Control values were subtracted and results expressed as percent β-hexosaminidase secretion as mean ± S.D. (*n* = 3). B, percent β-hexosaminidase secretion in 15 min from control or ionomycin-stimulated bone marrow–derived mast cells treated with the indicated concentrations of bexin-1 for 15 min are shown as mean ± S.D. (*n* = 3). *C*, RBL-2H3 cells expressing TNFα-pHluorin were left untreated or stimulated for 15 min with ionomycin. Cells were stained with Hoechst 33342, and cell surface fluorescence was quantitated by epifluorescence microscopy. Values for cells treated for 15 min with the indicated compound (20 μm) are shown as percent maximal fluorescence (mean ± S.D., *n* = 3). *D*, endocytosis was determined by the uptake of transferrin–Texas Red conjugate (Tf-Red). Cells were treated with 20 μm compounds for 15 min, followed by 15-min incubation with Tf-Red. Cells were washed, fixed, and imaged by confocal microscopy. Shown is a representative study of triplicate determinations (*, *p* < 0.05; **, *p* < 0.01). *E*, Alamar Blue was used to monitor cell viability upon exposure of live cells to inhibitors at the indicated concentrations for 5 h. The various compounds tested correspond to classes A (F5128-0085, bexin-1 and F5085-0061, bexin-5), B (F2927-0504), and D (F4448-0530) as representative of many compounds tested. For class F, we tested 13 compounds, with the most inhibitory members from the cell screen (F5024-0157 and F2590-0733) shown here.

To determine the specificity of inhibitory compounds for stimulated SG exocytosis, we utilized several orthogonal assays. The ionomycin-stimulated secretion of endogenous β-hexosaminidase was found to be strongly inhibited by bexin-1, -2, and -3 at 20 μm but not by bexin-5 or -6 (Fig. 3*A*). As anticipated, other compounds from the secondary screen also inhibited ionomycin-evoked β-hexosaminidase secretion, indicating that inhibition was not selective for SG cargo. Importantly, bexin-1 also inhibited ionomycin-stimulated β-hexosaminidase secretion in bone marrow–derived mast cells (Fig. 3*B*). Bexin-1 at 10 μm also inhibited IgE-stimulated β-hexosaminidase secretion from bone marrow–derived mast cells by 78%, whereas bexin-5 did not inhibit (IgE-stimulated, 18.7% ± 7.4%; IgE plus bexin-1, 4.1% ± 4.6%, *p* < 0.05; IgE plus bexin-5, 31.1% ± 11.3%). Thus, inhibition by bexins was not stimulus-dependent.

In another orthogonal assay, TNFα C-terminally fused with the pH-sensitive GFP variant ecliptic pHluorin was stably expressed in RBL-2H3 cells. Transmembrane TNFα with its C terminus oriented toward the acidic SG lumen was nonfluorescent in resting cells because of low pH quenching. SG exocytosis stimulated by ionomycin treatment resulted in increased fluorescence at the cell surface, detected by epifluorescence microscopy (Fig. 3*C*). The exocytosis of TNFα-pHluorin was fully inhibited by bexin-1, -2, and -3 but not by bexin-5 or -6 (Fig. 3*C*). As anticipated, other compound hits from the secondary screen also inhibited. Collectively, the orthogonal assays confirmed the strong inhibitory effects of bexin-1, -2, and -3 on regulated SG exocytosis independent of stimulus or SG cargo.

To determine whether other membrane trafficking pathways were affected by compounds, we monitored the uptake of fluorescent transferrin as a measure of endocytosis and endosomal trafficking. Neither bexin-1 nor bexin-5 at 20 μm had any effect on transferrin uptake by RBL-2H3 cells (Fig. 3*D*). By contrast, several other compounds from the secondary screen decreased transferrin uptake, indicating that they likely affect endocytosis. Last, compounds in serial dilutions from 2.5 to 40 μm exhibited little cytotoxicity, as assessed with the Alamar Blue reduction assay using 20 times longer incubations than those in secretion assays (Fig. 3*E*).

### Bexins act at late steps in SG exocytosis

**Figure 4.**
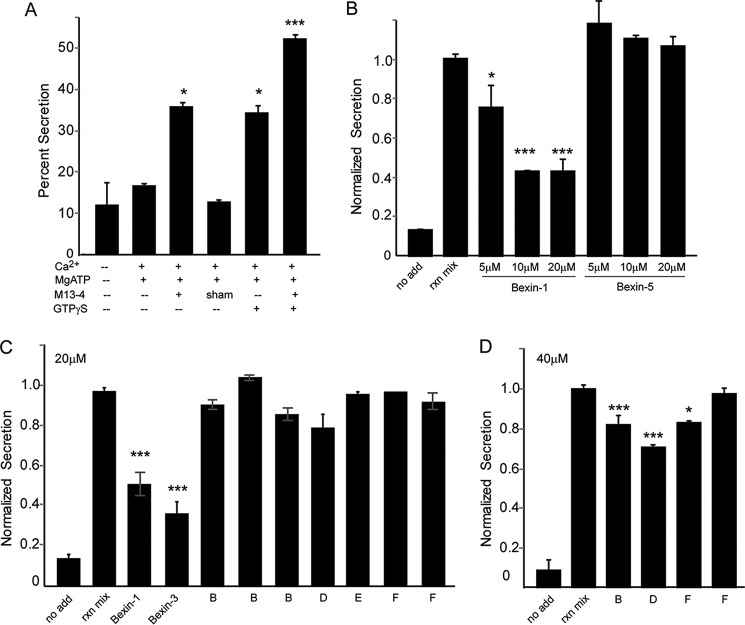
**Permeable RBL-2H3 cell assay for late steps in Ca^2+^-triggered secretory granule exocytosis.** *A*, RBL-2H3 cells stably expressing ANF-EGFP were permeabilized (see “Experimental procedures”), washed, and incubated under the indicated conditions for 15 min at 37 °C to detect the release of ANF-EGFP normalized to cell content (percent secretion). A representative study is shown, with mean ± S.D. for *n* = 3. Key additions that promoted ANF-EGFP release were 1 μm Ca^2+^, 2 mm MgATP, Munc13-4, and 10 μm GTPγS. *B*, bexins-1 and -5 were added to permeable cells at the indicated concentrations for 15 min prior to other additions (*add*) as in *A*, with mean ± S.D. for *n* = 4. *C*, the indicated compounds at 20 μm were added to permeable cells for 15 min prior to other additions as in *A*. A subset of representative compounds tested correspond to classes A (F5128-0085, bexin-1 and F2019-1958, bexin-3), B (F5075-5810, F2508-0544, F2508-1203), D (F5448-0530), E (F2297-0090), and F (F5024-0157, F2590-0733). *D*, the indicated compounds were added at 40 μm, corresponding to classes B (F2957-0504), D (F5448-0530), and F (F2590-0733 and F5024-0157). Representative studies are shown, with mean ± S.D. for n ≥ 4 indicated. *, *p* < 0.05; **, *p* < 0.01; ***, *p* < 0.001.

To further screen for inhibitors that act at late steps in Ca^2+^-dependent secretion, we employed an assay with permeable RBL-2H3 cells. Ca^2+^-triggered SG exocytosis can be reconstituted in washed permeable RBL-2H3 cells (17). The cells were permeabilized by a single pass through a ball homogenizer (>90% trypan blue–positive) and washed to remove soluble factors. Incubation of the permeable cells with Ca^2+^ and MgATP alone caused a modest release of ANF-EGFP (Fig. 4*A*), and addition of cytosol further stimulated secretion (17). The stimulatory effect of cytosol was fully replaced by the addition of 10 nm recombinant Munc13-4, as reported previously (17), or by cytosols from Munc13-4–expressing HEK293 cells but not by cytosols from sham-transfected HEK293 cells (Fig. 4*A*). The addition of nonhydrolyzable GTP analogs further increased Ca^2+^-triggered ANF-EGFP release (Fig. 4*A*), similar to previous reports for permeabilized mast cells (18, 19). Secretion in the complete permeable cell assay was comparable with that in intact ionomycin-stimulated RBL-2H3 cells (∼40–50% ANF-EGFP secreted within 10 min).

The reconstituted permeable cell assay reduced complexity and provided a direct assay for late steps in Ca^2+^-triggered SG exocytosis (17). Anticipating that small-molecule inhibitors acting at late steps would inhibit secretion in this assay, we assembled a sublibrary of 37 compounds representative of the chemical diversity in the secondary screen for classes A–E or the strongest inhibitors in the diverse class F (Fig. 1*H*). Representative results of testing these compounds in triplicate assays are shown in Fig. 4, *C* and *D* (see legend). Only three compounds, bexin-1, -2, and -3, inhibited Ca^2+^-stimulated secretion at 20 μm (Fig. 4*C* and data not shown). Bexin-1 inhibited secretion by 5 μm, whereas the less active bexin-5 failed to inhibit at 20 μm (Fig. 4*B*) or 40 μm (data not shown). None of the other compounds inhibited secretion in this assay except for three compounds that were very slightly inhibitory at 40 μm (Fig. 4*D*). Thus, inhibitory bexins uniquely appeared to act at late steps in Munc13-4–dependent SG exocytosis in permeable RBL-2H3 cells.

### Direct effects of bexins on the fusion machinery

Active bexins reliably inhibited Ca^2+^-dependent SG exocytosis in intact or permeable RBL-2H3 cells (Figs. 2–4). To determine whether bexins might directly interfere with priming or fusion events that follow SG translocation and docking, we utilized a liposome fusion assay that reconstitutes aspects of priming and fusion. Donor liposomes containing R-SNAREs with DiD and acceptor liposomes containing Q-SNAREs with DiI engage in concentration-dependent lipid mixing, measured as increased FRET. Previous studies showed that SNARE-dependent lipid mixing in liposomes is stimulated by accessory proteins such as Munc13-4 + Ca^2+^ (17), Munc18-1 (20), or synaptotagmin-1 fragment C2AB + Ca^2+^ (21). We used this highly reduced system to determine whether bexins act directly on any of the essential proteins involved in SG exocytosis.

**Figure 5.**
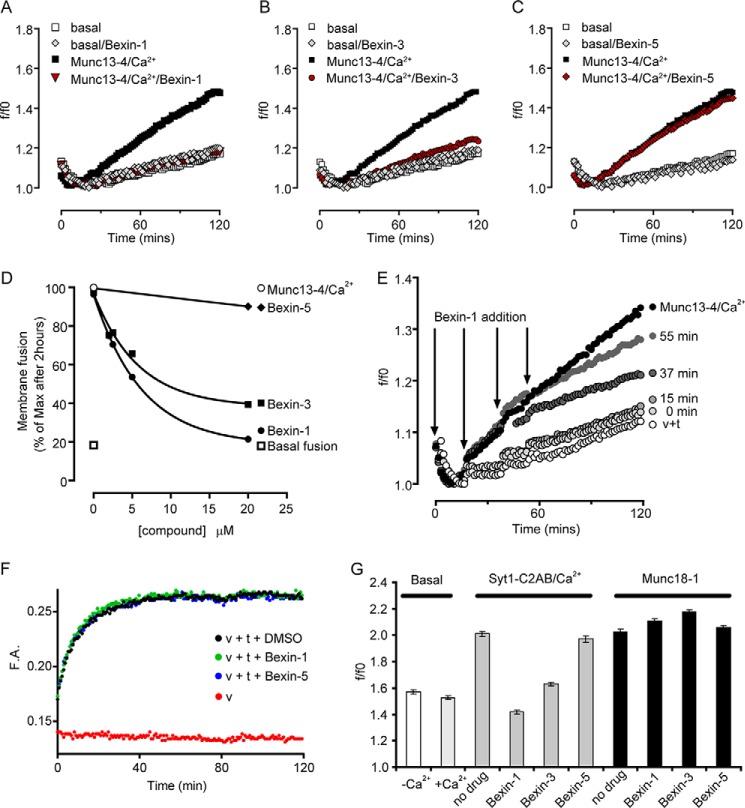
**Effects of compounds on SNARE-dependent lipid mixing.** *A–C*, liposome fusion assays (see “Experimental procedures”) were conducted at 37 °C without or with 1 μm Munc13-4 + 400 μm Ca^2+^ and with 20 μm bexin-1 (*A*), 20 μm bexin-3 (*B*), or 20 μm bexin-5 (*C*). *D*, the indicated concentrations of compounds bexins-1, -3, and -5 were tested in the liposome fusion assay with Munc13-4 + Ca^2+^. *E*, liposome fusion assays were conducted without (*open circles*) or with Munc13-4 + Ca^2+^ (*black circles*) or with bexin-1 added at 0, 15, 37, or 55 min (*shaded circles*). *F*, SNARE complex formation was detected by fluorescence anisotropy (see “Experimental procedures”) in the presence of DMSO or 20 μm bexins-1 or -5. *G*, liposome fusion assays were conducted with no additions (*Basal*) or with 1 μm Syt1-C2AB + 400 μm Ca^2+^ or 5 μm Munc18-1 plus 20 μm of the indicated compounds. The final extent of lipid mixing is shown as mean values with a range of duplicate determinations. Studies are representative of three or more experiments.

SNARE-dependent lipid mixing was accelerated by inclusion of Munc13-4 and Ca^2+^ (Fig. 5, *A–C*), as reported previously (17, 22). Bexin-1 and -3 fully inhibited Ca^2+^/Munc13-4–stimulated SNARE-dependent lipid mixing without affecting the basal rates of lipid mixing in the absence of Munc13-4 or Ca^2+^ (Fig. 5, *A* and *B*). Bexin-5, which was 20-fold less active than bexin-1 in intact or permeable cell assays, had little effect on Ca^2+^/Munc13-4-stimulated SNARE-dependent lipid mixing (Fig. 5*C*). Bexin-1 and -3 exerted inhibitory effects with IC_50_ values of ∼5 μm (Fig. 5*D*), which approximated the IC_50_ values observed in intact and permeable cell assays. In a delayed addition study (Fig. 5*E*), bexin-1 was found to inhibit Ca^2+^/Munc13-4–stimulated lipid mixing with minimal latency following its addition.

### Direct effects of bexins on Munc13-4

To further assess inhibition in the SNARE-dependent lipid mixing assay, we conducted additional studies on the bexins. It was unlikely that active bexins affected SNARE protein function because the basal rates of SNARE-dependent lipid mixing were unaffected. We monitored the rates of SNARE complex formation in an independent anisotropy assay to monitor binding of the fluorescent cytoplasmic R-SNARE VAMP2 to Q-SNARE syntaxin/SNAP25–containing liposomes (Fig. 5*F*). Neither bexin-1 nor bexin-5 inhibited SNARE complex formation in this assay, consistent with a lack of effect of bexins on SNARE function.

We assessed the selectivity of bexins as inhibitors of Ca^2+^/Munc13-4–dependent liposome fusion by testing other SNARE-binding regulatory proteins. Munc18-1 accelerates SNARE-dependent liposome fusion (20), but active bexin-1 and -3 as well as inactive bexin-5 failed to inhibit Munc18-1–stimulated lipid mixing (Fig. 5*G*). The results suggested that bexin-1 and -3 exert relatively selective inhibitory effects on Ca^2+^/Munc13-4. Munc13-4 contains N- and C-terminal Ca^2+^-binding C2 domains that bracket a central Munc homology domain–containing region. We also tested a protein fragment consisting of two tandem C2 domains (C2AB) from synaptotagmin-1. C2AB with Ca^2+^ stimulated SNARE-dependent lipid mixing, as reported previously (21) (Fig. 5*G*). Bexin-1 and, to a lesser extent, bexin-3 inhibited C2AB-stimulated lipid mixing, whereas bexin-5 was without effect.

**Figure 6.**
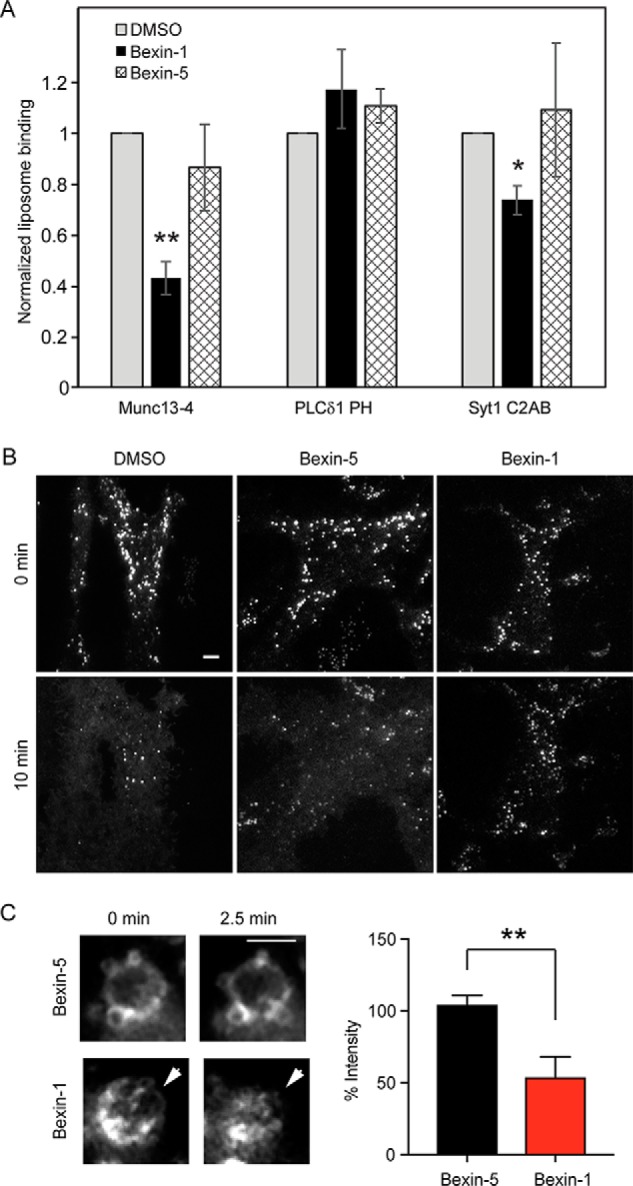
**Bexin-1 inhibits Munc13-4 membrane binding.** *A*, liposomes were incubated with 5 μm Munc13-4, PLCδ_1_PH, or Syt1 C2AB + Ca^2+^ in the presence of 0.1% DMSO or 20 μm bexin-1 or -5 prior to flotation and Western blotting to detect bound proteins. Mean ± S.D. (*n* = 3) values are shown (*, *p* < 0.05; **, *p* = 0.01). *B*, RBL-2H3 cells expressing EGFP-Munc13-4 were imaged by TIRF microscopy to detect Munc13-4 on docked SGs. Cells were treated with 0.1% DMSO or 10 μm bexin-1 or -5 for 5 min prior to ionomycin stimulation for 10 min at 37 °C. EGFP-Munc13-4 fluorescence transfers to the plasma membrane during exocytosis in DMSO- or bexin-5-treated but not in bexin-1-treated cells. *Scale bar* = 3 μm. *C*, EGFP-Munc13-4 dissociates from membranes upon treatment with bexin-1 but not bexin-5. EGFP-Munc13-4^+^ vacuole formation was stimulated in cells by treatment with ionomycin for 10 min at 37 °C, and 10 μm bexin-1 or bexin-5 was added. Vacuole fluorescence was recorded by epifluorescence during the 2.5-min incubation, and images were quantitated before and after compound addition. The *arrowheads* point to EGFP-Munc13-4 fluorescence on a vacuole surrounded by other smaller vacuoles. Values are mean ± S.D. (*n* = 3). **, *p* < 0.01. *Scale bar* = 3 μm.

These findings suggest that active bexins may target the C2 domains of Munc13-4 and possibly those of other proteins. Munc13-4 associates with acidic phospholipid-containing liposomes in a Ca^2+^-dependent manner through its C-terminal C2 domain (17, 22). Using a liposome flotation assay, we found that bexin-1, but not bexin-5, inhibited Ca^2+^-dependent Munc13-4 binding to liposomes (Fig. 6*A*). Bexin-1 also inhibited the binding of C2AB to a lesser extent. As a control, bexin-1 failed to affect the binding of a PH domain to liposomes. The results suggest that bexin-1, but not bexin-5, interferes with the C2 domain–mediated association of Munc13-4 with the membrane and possibly that of other C2 domain proteins.

Munc13-4 is essential for Ca^2+^-triggered SG-plasma membrane fusion in RBL-2H3 cells, and a fluorescent EGFP-Munc13-4 protein localizes to SGs (23). The Ca^2+^-triggered exocytosis of SGs can be monitored in TIRF microscopy as a transfer of Munc13-4 from SGs to the plasma membrane (Fig. 6*B*, *left panels*) (23). Bexin-1 fully blocked SG exocytosis measured in this manner (Fig. 6*B*, *right panels*), whereas bexin-5 was ineffective (Fig. 6*B*, *center panels*).

SGs in Ca^2+^-stimulated RBL-2H3 cells also undergo homotypic fusion to form vacuoles that fuse with the plasma membrane (23). We noted that the addition of bexin-1, but not bexin-5, caused dissociation of Munc13-4 from vacuole membranes, which was evident within 2.5 min of compound addition (Fig. 6*C*). These results were consistent with the idea that bexin-1 inhibits Ca^2+^- and Munc13-4–dependent membrane fusion by interfering with the association of Munc13-4 with the membrane.

### Bexins reduce SG–SG fusion and SG docking

**Figure 7.**
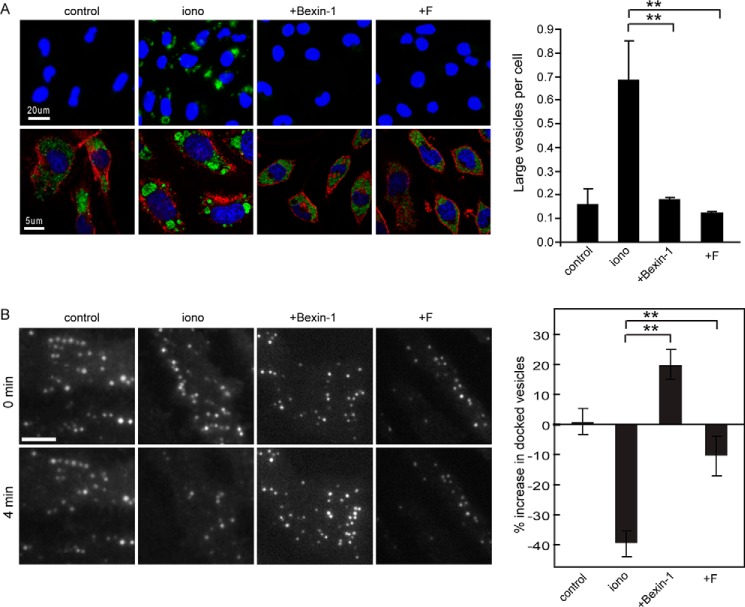
**Effect of bexin-1 on SG–SG fusion and SG-plasma membrane fusion.** *A*, cells expressing TNFα-pHluorin were incubated with DMSO vehicle, 20 μm bexin-1, or F2590-0733, as indicated, for 15 min and either left unstimulated (*control*) or stimulated with ionomycin (*iono*) for 15 min. *Top panels*, epifluorescence images of live cells, showing bright fluorescent puncta, indicating TNFα-pHluorin exocytosis. Cells were fixed, permeabilized, and stained with phalloidin-Alexa 568 and Hoechst 33342 (*bottom panels*). Fixation and permeabilization deacidifies SGs, allowing visualization of TNFα-pHluorin. Prior to stimulation, fluorescent puncta are small (*control*), whereas following stimulation (*iono*), larger puncta indicative of multigranular compound structures are evident. The number of large (>6 μm^2^) structures per cell was quantified (mean ± S.D.; *n* = 3; **, *p* < 0.01). *B*, SGs docked at the plasma membrane were monitored by TIRF microscopy in RBL-2H3 cells expressing ANF-EGFP. Cells were pretreated for 15 min with vehicle (*control*) or 20 μm bexin-1 or 20 μm F2590-0733. Individual SGs were resolved in TIRF as individual puncta and counted. Following incubation with ionomycin for 4 min or DMSO (*control*), SGs were counted, and differences with the 0-min values were determined. Differences between 0- and 4-min values were not evident in the absence of stimulation, whereas a net decrease in number was found for stimulated cells. The inhibition of secretion by bexin-1 resulted in a net increase in the number of docked SGs. Values represent mean ± S.D. (*n* = 16–34 cells). **, *p* < 0.01.

Bexin-1 was further tested on other aspects of SG function. As indicated above, a subset of SGs in RBL-2H3 cells engage in SG–SG fusion following ionomycin treatment to form larger multigranular vacuoles. Formation of these structures was blocked by Munc13-4 knockdown, revealing an additional activity of Munc13-4 in homotypic membrane fusion (23). In this work, we detected these compound structures with SG membrane–associated TNFα-pHluorin, which brightens in fixed cells. Although resting cells contained numerous small TNFα-pHluorin-positive SGs (Fig. 7*A*, *control*), ionomycin-treated cells contained larger (>1 μm in diameter) multigranular compound structures (Fig. 7*A*, *iono*). We quantitated the extent of SG–SG fusion by counting the number of large (>6 μm^2^) structures per cell. Bexin-1 at 20 μm was fully effective in blocking Munc13-4–dependent SG–SG fusion (Fig. 7*A*, +Bexin-1). A class F compound had a similar effect. Overall, these and previous data indicate that bexin-1 targets both Munc 13-4–dependent SG–plasma membrane and SG–SG fusion, which is consistent with previous findings that these Ca^2+^-dependent fusion events were blocked by knockdown of Munc13-4 (23).

The final steps in SG exocytosis involve translocation of SGs to the plasma membrane, followed by docking, priming, and fusion steps. To determine whether bexin-1 blocks translocation or the docking/priming/fusion steps, we monitored membrane-proximal SGs in ANF-EGFP–expressing cells by TIRF microscopy. Unstimulated cells contained SGs in the TIRF field that showed little movement in any direction, implying stable attachment or docking to the membrane (Fig. 7*B*, *control*). Upon stimulation with ionomycin, membrane-proximal SGs fused and disappeared from view (Fig. 7*B*, *iono*). In addition, new SGs appeared in the TIRF field during stimulation, with a subset of these undergoing fusion. Newly arrived SGs comprised ∼28% of all observed fusion events in stimulated cells. In a 4-min incubation with ionomycin, control cells showed a net ∼40% decrease in the number of SGs at the membrane (Fig. 7*B*, *iono*), reflecting a balance between new SG arrivals and SG fusion events. Compounds that inhibit SG fusion with the plasma membrane, but not SG translocation steps, would lead to an accumulation of SGs following stimulation. We assessed this in stimulated cells (≥16 cells per condition) by capturing live-cell images and counting the number of SGs visible in the TIRF field over the course of 4 min. Treatment with bexin-1 blocked SG fusion in this assay, resulting in SG accumulation at the membrane (Fig. 7*B*, +*Bexin-1*). By contrast, a class F compound blocked both SG fusion as well as the translocation of additional SGs to the plasma membrane (Fig. 7*B*, +*F*). Thus, consistent with previous studies, bexin-1 exerted inhibitory effects at a post-docking step of SG exocytosis.

## Discussion

There are many protein targets in the final steps of regulated or constitutive vesicle exocytosis, but few small-molecule inhibitors of vesicle exocytosis have been identified (6, 7). A small-molecule inhibitor of ERK1/2 that inhibits phosphorylation of EXO70, an exocyst subunit required for constitutive exocytosis, was found to inhibit constitutive secretion (24). In addition, a small molecule that directly binds EXO70 and inhibits constitutive secretion was characterized (25). SNARE-binding polyphenols were reported to inhibit liposome fusion *in vitro* and SG exocytosis in RBL-2H3 cells (26, 27). Inhibitors of Rab27a–JFC1 interactions were reported to inhibit regulated azurophilic granule exocytosis in neutrophils (28). These small-molecule targets represent only a small subset of the proteins active at late steps in vesicle exocytosis.

The high-throughput assay using intact RBL-2H3 cells was poised to detect inhibitors for steps in regulated secretion beyond Ca^2+^ mobilization or entry because ionomycin mediates direct Ca^2+^ entry into the cytoplasm. The late steps of Ca^2+^-triggered SG exocytosis have been elucidated at the molecular level in mast cells (11). R-SNARE proteins on SGs form complexes with Q-SNARE proteins on the plasma membrane to mediate docking, priming, and fusion steps (1, 3). SNARE complex formation is promoted by priming factors from the Sec1/Munc18 and Munc13/CAPS protein families (2, 3) corresponding to Munc18-1/2 and Munc13-4, respectively, in RBL-2H3 cells (11, 17). Munc13-4 is expressed at high levels in RBL-2H3 cells compared with PC12 cells and may be a major target for inhibitors. Rab proteins on SGs play a role in targeting priming factors, and Rab27 binds Munc13-4 for regulated SG exocytosis in RBL-2H3 cells (29). Final Ca^2+^-triggered fusion steps are mediated by synaptotagmins in other cell types, but these have not been identified for SG exocytosis in RBL-2H3 cells (11). Any of these proteins are potential targets for inhibitor action at late steps in SG exocytosis.

We utilized a series of progressively informative assays to discover novel inhibitors of mast cell degranulation and to identify a molecular target for a set of structurally related inhibitors. Our high-throughput and secondary screen with intact RBL-2H3 cells identified 129 compounds that inhibited secretion by ≥50% inhibition at 4 μm. 38 of these contained a 2-aminobenzothiazole scaffold, which we termed bexins. In addition, two compounds in the library that were not inhibitory in the screen served as structurally related controls (bexin-5 and -6). In assays with permeable RBL-2H3 cells or in SNARE-dependent lipid mixing, bexin-1, -2, and -3 prevailed as key inhibitors, whereas bexin-5 and -6 lacked inhibitory activity.

Benzothiazole molecules with different ring substituents exert a large variety of biological effects, including anti-cancer, anti-inflammatory, and anti-microbial activities (30). Optimized inhibitors in this class commonly harbor bulky constituents at the 5- or 6- positions on the benzothiazole ring. For example, 2-aminobenzothiazoles that target phosphoinositide 3-kinase contain groups at the 6-position that help align the purine-like benzothiazole ring in the ATP-binding site of phosphoinositide 3-kinase (31). By contrast, the bexins identified in this study as active inhibitors of regulated secretion in RBL-2H3 and mast cells lack bulky substituents on the benzothiazole ring and are extended at the 2-position via amino substitutions. Extensive searches for bexin-1, -2, and -5 in the PubChem, SciFinder, and ChEMBL databases failed to identify any bioactivities reported for these molecules. By contrast, bexin-3 with its phenylsulfonylhydroquinoline substituent was found to have numerous bioactivities in PubChem. None of the bexins were pan assay interference compounds (32). Thus, this study assigns novel biological activity to bexin-1 and -2.

Permeable RBL-2H3 cells preserve late steps of regulated SG exocytosis, and studies showed that Munc13-4 was required for optimal Ca^2+^-triggered secretion (17), indicating that Munc13-4 is a potential target for the bexins. This was explicitly tested in a liposome fusion assay that recapitulates physiological properties of Munc13-4 (17). Bexin-1 and -3 inhibited the Ca^2+^- and Munc13-4–dependent promotion of SNARE-dependent lipid mixing. By contrast, these compounds did not affect Munc18-stimulated liposome fusion. Importantly, bexin-1 and -3 did not affect basal fusion, which indicated a lack of effect on SNARE complex formation, which was confirmed in an independent SNARE protein assembly assay. From these studies, we conclude that Munc13-4 is a direct target for inhibitory bexins.

Munc13-4 interacts with both SNARE proteins as well as with acidic phospholipid-containing membranes in a manner regulated by or mediated by the two Ca^2+^-binding C2 domains of the protein (17, 22, 33). We found that bexin-1 inhibited the Ca^2+^-stimulated binding of Munc13-4 to acidic phospholipid-containing liposomes. Two previous studies ascribed the Ca^2+^-dependent membrane binding properties of Munc13-4 to its C-terminal C2-domain (17, 22), suggesting that bexin-1 interferes with a C2 domain–mediated function of Munc13-4. Moreover, we found that treatment of intact RBL-2H3 cells with bexin-1, but not bexin-5, strongly inhibited Ca^2+^-triggered membrane fusion while concomitantly dissociating Munc13-4 from cellular membranes. This links the inhibitory effects of bexin-1 on membrane fusion to its ability to interfere with Munc13-4–membrane association. Inhibition of SG–SG fusion and SG-plasma membrane fusion with accumulation of unfused SGs by bexin-1 is similar to the impact of Munc13-4 knockdown (23), indicating that bexin-1 inhibits all of the characterized Munc13-4–dependent fusion events in RBL-2H3 cells.

We attribute the inhibitory action of bexins to interference with Munc13-4 C2 domain interactions with the membrane. An open question is the selectivity of the bexins for cellular effects. Stimulation of SNARE-dependent lipid mixing by synaptotagmin C2AB and C2AB binding to liposomes was also inhibited by bexin-1 to some extent. However, the soluble synaptotagmin C2AB protein differs substantially from the native membrane protein in lipid mixing assays, so it is difficult to extrapolate the liposome studies into a cellular context (34). Bexins were only very weakly inhibitory in PC12 cells, where synaptotagmins-1 and -9 are essential for regulated SG exocytosis (35). In RBL-2H3 cells, the identity of a Ca^2+^-binding synaptotagmin required for regulated SG exocytosis remains unclear (36). Thus, we attribute the inhibitory actions of bexins in RBL-2H3 cells to Munc13-4 inhibition. However, small molecules with micromolar efficacy exhibit off-target effects (37), so the possibility that bexins also inhibit other C2 domain proteins cannot be currently assessed. Preliminary studies indicated that bexin-1 affects cell shape and inhibits Cdc42 GTP exchange in RBL-2H3 cells, which might be mediated through inhibition of the intersectin 1L C2 domain. However, acute treatment with secramine, a Cdc42 inhibitor (38), did not affect RBL-2H3 secretion. Whether C2 domain proteins other than Munc 13-4 are inhibited by the bexins remains to be determined.

The mechanism of inhibition by bexin-1 of Munc13-4 function will need to be clarified in future studies. A key target of bexin-1 may be the Munc13-4 C2 domain–membrane interface. C2 domains consist of eight-stranded β sandwich structures with four β-strand sheets on each side of the sandwich (39). A class of 2-arylbenzothiazoles that includes thioflavin-T has been extensively studied for binding to β sheet structures such as those in β-amyloid fibrils (40–43). This suggests the possibility that the benzothiazole ring of bexin-1 may interact with the β sheet structures in C2 domains. The amidopyrazole ring in bexin-1, which extends from the benzothiazole ring, is lipophilic and might interact with the membrane or within the C2 domain to interfere with Ca^2+^-dependent Munc13-4 C2 domain membrane interactions. The inference that bexin-1 acts at a C2 domain–membrane interface will need to be tested in future studies.

## Experimental procedures

### Plasmids and antibodies

ANF-EGFP, a kind gift from E. Levitan (University of Pittsburgh), was subcloned into lentivirus plasmids. TNFα-pHluorin plasmids were constructed from TNFα(1–79) plasmids generously provided by M. Olszewski (Warsaw, Poland). GFP-Munc13-4 constructs were described previously (23). Plasmid constructs pTW34 to express rat syntaxin-1A with N-terminally His-tagged mouse SNAP-25B and pTW2 to express C-terminal His-tagged mouse VAMP-2 were provided by J. E. Rothman (Yale University, New Haven, CT) and T. Weber (Mount Sinai School of Medicine, New York, NY), and proteins were purified as described previously (44) by nickel-nitrilotriacetic acid (Qiagen) chromatography. His6-tagged human Munc13-4 protein produced in insect Sf9 cells was purified on nickel-nitrilotriacetic acid–agarose and further purified by Mono Q anion exchange chromatography (GE Healthcare). GST-C2AB (synaptotagmin-1) was prepared as described previously (45). Monoclonal antibodies to RMCP2 were obtained from Moredun Scientific (Penicuik, Midlothian, Scotland). Rabbit polyclonal VMAT-2 antibody was generously provided by A. Ruoho (University of Wisconsin, Madison).

### Cell culture and immunocytochemistry

RBL-2H3 cells (CRL-2256, American Type Culture Collection, Manassas, VA) were cultured in in minimal essential medium (Invitrogen) with 15% (v/v) added fetal bovine serum in a 5% CO_2_ incubator at 37 °C. Stable clones (isolated from single cells by limiting dilution) were generated by lentiviral transduction with ANF-EGFP, subcloned into a pWPXL expression vector, and packaged with the psPAX2 packaging vector and pMD2.G vesicular stomatitis virus G-protein (VSV-G) envelope vector from Addgene (Cambridge, MA). Bone marrow–derived mast cells were cultured from B6 mice as described previously (46). Mouse procedures were reviewed and approved by the University of Wisconsin, Madison Institutional Animal Care and Use Committee. PC12 cells were cultured as described previously (47) using standard methods. ANF-EGFP–expressing PC12 cell lines were established by lentiviral transduction with puromycin selection with cloning by dilution. COS-1 cells were cultured in Dulbecco's modified Eagle's medium supplemented with 10% fetal bovine serum in a 5% CO_2_ atmosphere. Cells were transfected by electroporation using conditions described by Van den Hoff *et al.* (53) with 50 μg of plasmid DNA/10^7^ cells. Immunofluorescence studies were conducted on cells washed in PBS, fixed for 10 min in 4% paraformaldehyde, and permeabilized for 15 min in PBS with 1% TritonX-100. Following primary and secondary antibody incubations, cells were stained with 5 μm Hoechst 33342 dye to detect nuclei. Transferrin uptake studies were conducted with transferrin–Alexa Fluor 647 conjugates (Tf-Red) obtained from Life Technologies. Cell viability assays were conducted with Alamar Blue (Thermo Fisher Scientific) according to the vendor's protocol.

### Microscopy

For fluorescence microscopy, glass coverslips were coated with 0.1 mg/ml poly-d-lysine and 30 μg/ml bovine fibronectin at 37 °C (Sigma-Aldrich). Confocal images were acquired either on a Nikon C1 laser-scanning confocal microscope with a ×60 oil immersion objective with NA 1.4 or with a Nikon A1R+ confocal system using GaAsP detectors. Images were deconvolved, processed, and analyzed by NIS software (Nikon, Tokyo, Japan). TIRF images were acquired on a Nikon evanescent wave imaging system on a TE2000-U inverted microscope with an Apo TIRF ×100 NA 1.45 objective lens at 4 Hz with a CoolSNAP-ES digital monochrome charge-coupled device (CCD) camera system (Photometrics, Woburn, MA) controlled by Metamorph software (Universal Imaging, West Chester, PA). Alternatively, TIRF images were acquired on a Nikon Eclipse Ti microscope controlled by NIS Element software with image capture by an iXon Ultra EM-CCD camera through a Nikon APO ×100 NA 1.49 objective. Time-lapse imaging for live cells was conducted in a humidified imaging chamber maintained at 37 °C (Tokai Hit, Shizuoka-ken, Japan). Image analysis was done using Fiji/ImageJ (42) or NIS Element software.

### Cell secretion assays

Live-cell secretion assays with ANF-EGFP–expressing RBL-2H3 and PC12 cells were performed in Tyrode buffer (130 mm NaCl, 5 mm KCl, 1 mm MgCl_2_, 20 mm HEPES, 5.6 mm glucose, and 0.5 mg/ml BSA (pH 7.4)). Cell monolayers were washed, and compounds were added 15 min prior to the addition of buffer (control) or 2 μm ionomycin for 15 min at 37 °C. Fluorescence at 488 nm excitation of ANF-EGFP in buffer overlying cells was determined after low-speed centrifugation to remove detached cells, and attached cells were solubilized in 1% Triton X-100 for calculating percent secretion. The secretion of β-hexosaminidase by RBL-2H3 cells was determined in a similar format by monitoring enzymatic activity in supernatants and solubilized cells using the chromogenic substrate 4-nitrophenyl *N*-acetyl-β-d-glucosaminide (pNAG) as described previously (23). For antigen stimulation, cells were primed with 200 ng/ml anti-2,4-dinitrophenol monoclonal IgE in growth medium overnight and subsequently washed with Tyrode buffer and incubated with 50 ng/ml 2,4-dinitrophenol–human serum albumin in Tyrode buffer containing 2 mm CaCl_2_ for 20 min.

### High-throughput screening

Automated liquid handling and drug library screening were conducted at the University of Wisconsin Small Molecule Screening Facility. Drugs were applied by pin transfer, and automated liquid transfer was performed by Biomek FX. The compound library (LC II) from Life Chemicals was provided as 4 mm stocks in DMSO by the University of Wisconsin Small Molecule Screening Facility. Compounds annotated in this study are given as the external identifier given by Life Chemicals that are searchable in PubChem.

### Permeable cell secretion assays

Permeable RBL-2H3 and PC12 cell secretion assays were conducted with cells permeabilized by passage through an appropriately fitted ball homogenizer (48). A stable clone of RBL-2H3 rat mast cells expressing ANF-EGFP was generated by lentiviral transduction. Targeting of ANF-EGFP to secretory granules and cosecretion with the endogenous granule marker β-hexosaminidase were confirmed. Permeable RBL-2H3 secretion assays were conducted at 30 °C in buffers containing 0.05 m HEPES, 0.12 m potassium glutamate, 0.002 m EGTA, and 0.1% BSA (pH 7.2) adjusted to 3 μm free Ca^2+^ and containing 2 mm MgATP and 10 μm GTPγS. The cytosol used in the RBL-2H3 cell assays was prepared from COS-1 cells, either WT (sham) or expressing Munc13-4, by homogenization in ice-cold 0.02 m HEPES (pH 7.5), 0.002 m EGTA, 0.001 m EDTA, 0.001 m DTT, 0.0001 m phenylmethylsulfonyl fluoride, and 0.5 μg/ml leupeptin using multiple passes in a ball homogenizer, followed by centrifugation at 30,000 × *g* for 30 min and 100,000 × *g* for 90 min. In some assays, purified Munc13-4 was used for comparison. After incubation, RBL-2H3 cells were centrifuged at 650 × *g*, and the supernatant and 1% Triton X-100–solubilized pellet fractions were assessed for fluorescence in a plate reader (Infinite F500, Tecan Group Ltd.) to determine the percentage of ANF-EGFP secreted.

### Liposome fusion assays, liposome binding, and SNARE complex formation

Lipid-mixing fusion assays were conducted as described previously (17, 23, 49). Lipid mixing was reported as FRET between DiI-containing VAMP-2 liposomes and DiD-containing syntaxin-1A/SNAP-25 liposomes. The standard assay used 0.45 mm of acceptor and 0.225 mm of donor liposomes in a total volume of 75 μl of reconstitution buffer without glycerol supplemented with 0.1 mm EGTA. Munc13-4 protein was added at the concentrations indicated in the figure legends. CaCl_2_ was added to achieve the free Ca^2+^ indicated in the figures. Control reactions were prepared for all conditions by substituting syntaxin-1A/SNAP-25 acceptor liposomes with protein-free liposomes to detect non-SNARE-mediated lipid mixing. Reactions were assembled on ice and mixed before addition to 96-well FluoroNunc plates. Lipid mixing was observed as an increase in FRET from DiI to DiD labels by measuring DiD fluorescence at 700 ± 5 nm during DiI excitation at 514 ± 5 nm every 90 s over 2 h at 35 °C using the SPECTRAmax GEMINI-XS spectrofluorometer (Molecular Devices, Sunnyvale, CA). Results are expressed as the ratio of fluorescence at time x/minimum fluorescence measured over 2 h. SNARE complex formation was determined by anisotropy utilizing syntaxin-1A/SNAP25 acceptor liposomes incubated with a cytoplasmic domain of VAMP2 labeled at residue 28 with Alexa Fluor 488, similar as described previously (50).

Liposomes were generated by resuspending a nitrogen gas–dried lipid film containing 1.5 μmol of POPC/DOPS/PI(4,5)P_2_ in an 87:12:1 mole ratio in 500 μl of elution buffer (25 mm HEPES-KOH (pH 7.4), 100 mm KCl, 50 mm imidazole-OAc (pH 7.4), and 1.0% β-octylglucoside) for 30 min, diluted 2-fold with reconstitution buffer (25 mm HEPES (pH 7.4), 100 mm KCl, 10% glycerol, and 1 mm DTT) by dropwise addition, and dialysis overnight against reconstitution buffer containing Bio-Beads (Bio-Rad). Lipid mixtures were spiked with 2 μl of [^3^H] 1,2-dipalmitoyl phosphatidylcholine (∼2 × 10^5^ cpm/nmol, DuPont) to assess lipid recoveries and to standardize co-flotation assay reactions. The liposomes were purified by buoyant density centrifugation on an Accudenz step gradient (3 ml at 40%, 2 ml at 30%, and 0.5 ml at 0% in reconstitution buffer) at 45,000 rpm for 4 h in an SW50.1 rotor (Beckman Coulter). Liposome co-flotation assays were performed as described previously (44) with modifications. Liposomes were incubated with 5 μm Munc13-4, GST-Syt1C2AB, or PLCδ_1_PH-GFP and, where indicated, plus 400 μm calcium for 30 min at room temperature in 75 μl of reconstitution buffer. Compounds were added to reactions after protein for a final concentration of 20 μm and 0.6% v/v DMSO. In reactions without compounds, DMSO was added to yield a final concentration of 0.6% v/v. 75 μl of 80% Accudenz was added to the binding reaction to yield a final concentration of 40% Accudenz. 30% Accudenz and reconstitution buffers were layered on top and centrifuged for 4 h in an SW50.1 rotor at 45,000 rpm. Accudenz gradient layers were supplemented with calcium and compounds to provide concentrations equivalent to those in the binding reactions. Either the floated fraction or all fractions were collected, run on SDS-PAGE, and analyzed by Western blotting or SYPRO Ruby staining. ImageJ/Fiji software (National Institutes of Health) was used to quantify protein bands. Constructs encoding PLCδ_1_PH-GFP were generously provided by T. Balla (National Institutes of Health) and GST-Syt1C2AB by E. Chapman (University of Wisconsin, Madison, WI) for protein production in *Escherichia coli* as described previously (51, 52).

## Author contributions

S. B. and T. F. J. M. conceptualization; S. B., D. J. J., M. Q. S., J. E., S. S. W., E. K.-G., E. C., R. Q., and J. A. K. data curation; S. B., D. J. J., M. Q. S., J. E., S. S. W., E. K.-G., E. C., R. Q., and J. A. K. formal analysis; S. B. and T. F. J. M. funding acquisition; S. B., D. J. J., M. Q. S., J. E., S. S. W., E. K.-G., and E. C. investigation; S. B., D. J. J., M. Q. S., J. E., S. S. W., E. K.-G., and E. C. methodology; S. B., D. J. J., and T. F. J. M. writing-original draft; S. B., D. J. J., and T. F. J. M. writing-review and editing.

## Footnotes

7 The abbreviations used are:

SG: secretory granule
SNARE: soluble *N*-ethylmaleimide-sensitive factor attachment protein receptor
TNF: tumor necrosis factor
ANF: atrial natriuretic factor
EGFP: enhanced GFP
bexin: benzothiazole exocytosis inhibitor
PH: pleckstrin homology
GST: glutathione *S*-transferase
NA: numerical aperture
GTPγS: guanosine 5′-3-*O*-(thio)triphosphate
Tf-Red: transferrin–Texas Red
DiD: 1,1′-dioctadecyl-3,3,3′,3′-tetramethylindodicarbocyanine, 4-chlorobenzenesulfonate salt
DiI: 1,1′-dioctadecyl-3,3,3′,3′-tetramethylindocarbocyanine
TIRF: total internal reflectance fluorescence
POPs: 1-palmitoyl-2-oleoyl-*sn*-glycero-3-phosphocholine
DOPs: 1,2-dioleoyl-*sn*-glycero-3-phospho-l-serine; brain l-α-phosphatidylinositol-4,5-bisphosphate.
